# PD43 - Body fat mass is positively associated with pediatric asthma

**DOI:** 10.1186/2045-7022-4-S1-P43

**Published:** 2014-02-28

**Authors:** George V Guibas, Yannis Manios, Paraskevi Xepapadaki, George Moschonis, Nikolaos Douladiris, Christina Mavrogianni, Nikolaos G Papadopoulos

**Affiliations:** 1 Allergy Department, 2nd Pediatric Clinic, University of Athens, Athens, Greece; 2 Department of Nutrition and Dietetics, Harokopio University, Athens, Greece

## Background

Prevalence of pediatric overweight/obesity and pediatric asthma has been on the rise, with both conditions currently reaching epidemic proportions. Their concurrent rise alludes to potentially common characteristics of their pathophysiologic mechanisms; furthermore, excess fat mass may facilitate asthma induction via obesity-related inflammatory mediators, oxidant stress and mechanical chest restriction. However, although such a link is well-documented in adults, it is not yet established in children. We thus opted to investigate into a potential adiposity/asthma association in a cross-sectional, population-based study in preschoolers, by using several indices to assess fat mass.

## Methods

Wheeze ever/in the last 12 months (current) and physician-diagnosed asthma were recorded from questionnaires filled in by the parents of 2015 children aged 9-13. Perinatal data was collected from their medical records and the questionnaires; anthropometric measurements and bioelectric impedance analysis (BIA) were conducted. Logistic regression models were build in the Statistical Package for Social Sciences (SPSS version 20.0), with the wheeze/asthma variables as main outcomes. A two-tailed p value less that 0.05, was considered statistically significant.

## Results

Physician-diagnosed asthma correlated with z scores of BMI (OR=1.17 95%CI=1.05-1.31, p=0.005), waist circumference (OR=1.16 95%CI=1.03-1.32, p=0.017), waist-to-height ratio (OR=1.18 95%CI=1.04-1.34, p=0.009), sum skinfold thickness at 4 sites (biceps, triceps, subscapular, suprailliac) (OR=1.21 95%CI=1.07-1.38, p=0.002) and bioelectric impedance-derived percentage fat mass (OR=1.23 95%CI=1.07-1.40, p=0.003), following adjustment for several potential confounding factors (prenatal smoking, gestational age, birth weight, gender, parity, breastfeeding, passive smoking at home and parental educational level). Parental-reported current/ever wheeze was not associated with fat mass.

## Conclusions

Body fat mass is positively linked to pediatric asthma prevalence in preadolescent children.

